# Draft genome sequences of the pathogenic fungi *Scedosporium aurantiacum* and *Scedosporium apiospermum* from clinical isolates

**DOI:** 10.1128/mra.00896-25

**Published:** 2025-12-17

**Authors:** Samuel T. Montgomery, Hamideh Rezaee, Nicolau Sbaraini, Joshua Iszatt, Liza Mantjani, Sarah E. Kidd, Yit-Heng Chooi, Luke W. Garratt

**Affiliations:** 1 Wal-Yan Respiratory Research Centre, Kids Research Institute Australia, The University of Western Australia 2720, Perth, Western Australia, Australia; 2 Centre for Child Health Research, The University of Western Australia 2720, Perth, Western Australia, Australia; 3 School of Molecular Sciences, The University of Western Australia, Perth, Western Australia, Australia; 4 National Mycology Reference Centre, Microbiology & Infectious Diseases, SA Pathology 10107, Adelaide, South Australia, Australia; 5 School of Biological Sciences, Faculty of Sciences, Engineering and Technology, University of Adelaide 1066, Adelaide, South Australia, Australia; University of California Riverside, Riverside, California, USA

**Keywords:** *Scedosporium*, genomes, filamentous fungi

## Abstract

*Scedosporium* species are filamentous fungi with inherent broad antifungal resistance that pose opportunistic infection threats. We present draft genome assemblies of *S. aurantiacum* (11 contigs) and *S. apiospermum* (9 contigs), derived from Oxford Nanopore sequencing of one Australian clinical isolate each.

## ANNOUNCEMENT

In the 2022 World Health Organization Fungal Priority Pathogen List (1), *Scedosporium* species ranked fourth for research needs. Genomic variation of *Scedosporium* is poorly described (2). Here, deidentified clinical isolates of *S. aurantiacum* (strain ID-16-90050374; otomycosis, Darwin, Australia) and *S. apiospermum* (strain ID-17-90028250; subcutaneous mycosis, Melbourne, Australia) were obtained from the National Mycology Reference Centre (Adelaide, Australia) for research, as permitted under the National Statement on Ethical Conduct in Human Research. Isolates were cultivated in Sabouraud Dextrose liquid media at 37°C under aerobic conditions with agitation for 4–5 days. The mycelial biomass was collected by filtration through Kimwipes, ground to a fine powder using mortar and pestle with liquid nitrogen, and high-molecular-weight DNA extracted using phenol/chloroform (3). Indexed paired-end libraries for short-read sequencing were prepared using Nextera XT DNA Library Prep (Illumina, USA) under manufacturer’s instructions, quality assessed using a Bioanalyzer 2100 (Agilent Technologies, USA), and whole-genome sequencing performed on the Illumina HiSeq 2500 platform with 100 bp paired-end reads. For long-read sequencing, Oxford Nanopore libraries prepared using the Native Barcoding Kit (NBD-114-96), with no size selection or shearing, were sequenced on a PromethION flow cell on a P2 Solo. Data were basecalled using dorado (v0.5.3) using the dna_r10.4.1_e8.2_400bps_sup@v4.3.0 model.

Long-read sequencing yielded 3,469,118 and 1,864,815 reads for *S. aurantiacum* and *S. apiospermum,* respectively, with an N50 value of 12.50 kb and 11.91 kb and read lengths up to 133 kb (Table 1). Data were filtered using chopper (v0.10.0) (4) to remove reads <1 kb and <Q20, and sequencing adaptors were trimmed using porechop_abi (v0.5.0) (5). Filtered reads were assembled using flye (v2.9.6) (6) using default parameters specifying --scaffold, --asm-coverage 50, and --read-error 0.01. The resulting *de novo S. aurantiacum* and *S. apiospermum* assemblies are 39,272,335 bp and 43,356,729 bp long, comprising 11 and 9 contigs, respectively (Table 1). Short reads, filtered with AAFTF (v0.2.2) and default parameters (7), were used to polish contigs using both PyPOLCA (v0.3.1) (8, 9) with the --careful flag and polypolish (v0.6.0) (10). Reads were mapped back to the final assemblies using minimap2 (v2.2.29) (11) with long reads using the lr:hq preset and short reads using the sr preset and quality metrics obtained using qualimap (v2.3) (12). Telomeric repeats (AACCCT) were identified using tidk (v0.2.65) (13), with *S. aurantiacum* containing telomeric repeats on both ends of eight contigs and on a single end of two contigs, while the *S. apiospermum* assembly contains telomeric repeats on both ends of eight contigs and a single end of one contig. We used funannotate2 (v25.7.1) (14) and helixer (v0.3.5) (15) for repeat masking, gene prediction, and annotation using all default evidence-guided and *ab initio* predictions. Protein evidence for model training was derived using *Sordariomycetes* as the seed data for BUSCO searches.

**TABLE 1:** Summary statistics for the *Scedosporium aurantiacum* and *Scedosporium apiospermum* genome assembly and annotation

	*S. aurantiacum* (ID-16-90050374)	*S. apiospermum* (ID-17-90028250)
Nanopore reads (*n*)	3,469,118	1,864,815
Read N50 (bp)	12,498	11,905
Median read *Q* score	19.0	18.9
Illumina reads (*n*)	40,364,656	40,576,204
Assembly size (bp)	39,272,335	43,389,729
Contigs (*n*)	11	9
Contig N50 (bp)	4,772,094	5,346,190
GC %	49.82	50.43
BUSCO complete %	95.5	95.3
Repeat sequences (%)	6.33	4.74
Long-read mapping (%)	99.87	99.99
Long-read mean coverage	745.58	324.28
Short-read mapping (%)	83.14	99.25
Short-read mean coverage	127.89	139.04
Predicted genes (*n*)	10,523	12,291
Predicted CDS (*n*)	10,244	12,012
GenBank BioSample accession	SAMN48578032	SAMN48578033
Genbank Assembly accession	GCA_052249765.1	GCA_052249755.1

## Data Availability

All data are available under the National Center for Biotechnology Information BioProject number PRJNA1249256. Assembled genomes are also available at https://doi.org/10.5281/zenodo.17538694. Long-read sequencing data are available at SRX30026839 (S. aurantiacum) and SRX30026840 (S. apiospermum). Short-read sequencing data are available at (S. aurantiacum) SRX30026841 and SRX30026842 (S. apiospermum).
